# Analytical Study Showing a False-Negative Limitation of Deletion-Based Newborn Screening for Spinal Muscular Atrophy Using a Compound Heterozygous *SMN1* Control

**DOI:** 10.3390/ijns12030071

**Published:** 2026-08-31

**Authors:** Yuichi Abe, Sato Suzuki-Muromoto, Motomichi Kosuga, Tetsuya Isayama, Go Tajima, Yasuhito Aoki, Nobuyuki Ishige, Yushi Ito

**Affiliations:** 1 Division of Neurology, National Center for Child Health and Development, Tokyo 157-8535, Japan; 2 Center for Genetic Medicine, National Center for Child Health and Development, Tokyo 157-8535, Japan; 3 Division of Medical Genetics, National Center for Child Health and Development, Tokyo 157-8535, Japan; 4 Division of Neonatology, National Center for Child Health and Development, Tokyo 157-8535, Japan; 5 Division of Neonatal Screening, Research Institute, National Center for Child Health and Development, Tokyo 157-8535, Japan; 6 Diagnostics Division, Sekisui Medical Co., Ltd., Tokyo 103-0027, Japan; 7 Division of Newborn Screening, Tokyo Health Service Association, Tokyo 162-0842, Japan; 8 Department of Neonatology, Sanno Hospital, Tokyo 107-0052, Japan

**Keywords:** spinal muscular atrophy, newborn screening, *SMN1*, compound heterozygous variant, false-negative result, dried blood spot

## Abstract

Newborn screening (NBS) facilitates the presymptomatic diagnosis and early treatment of spinal muscular atrophy (SMA). An analytical study was conducted to evaluate the performance and limitations of a real-time polymerase chain reaction-based SMA-NBS assay. Dried blood spot specimens collected from 21 newborns and three patients with genetically confirmed SMA were analyzed. The SMA controls included two patients with homozygous *SMN1* deletion and one with compound heterozygous *SMN1* variants. *SMN1* exon 7 copy numbers were quantified and interpreted. All newborn participants had *SMN1* copy numbers above the screening cutoff value of 668 copies/µL and were classified as screening-negative. The two SMA controls with homozygous *SMN1* deletion showed no detectable *SMN1* amplification, which is consistent with positive screening results. In contrast, the compound heterozygous SMA control exhibited a mean *SMN1* value of 7330 copies/µL, which was more than tenfold above the screening cutoff and would therefore have been classified as screening-negative; this value also fell within the range observed in the newborn cohort. This patient had a negative NBS result at birth despite subsequently developing genetically confirmed SMA. The SMA-NBS assay accurately detected patients with homozygous *SMN1* deletion. However, it did not identify patients with compound heterozygous *SMN1* variants. In conclusion, deletion-based SMA-NBS has an inherent false-negative limitation, and a negative screening result does not completely exclude an SMA diagnosis.

## 1. Introduction

Spinal muscular atrophy (SMA) is an autosomal recessive neuromuscular disorder characterized by progressive muscle weakness caused by the degeneration of alpha motor neurons in the anterior horn of the spinal cord. The incidence of SMA is approximately 1 in 6000–10,000 live births, making it one of the most common genetic causes of infant mortality [1,2,3]. According to age at onset and the highest motor milestone achieved, SMA is clinically classified into types 0–IV. Patients with the most severe types of SMA (types 0 and I) typically develop the condition during the prenatal period or early infancy, and they cannot achieve independent sitting [2].

The survival motor neuron 1 gene (*SMN1*), located on chromosome 5q13.2, encodes the survival motor neuron protein, which is essential for motor neuron function. Homozygous deletion of *SMN1* exon 7 accounts for approximately 95% of SMA cases. Meanwhile, the remaining cases involve compound heterozygous variants comprising a deletion on one allele and an intragenic pathogenic variant on the other allele [1,4,5]. Most patients carry homozygous *SMN1* deletions. Thus, molecular diagnosis can generally be achieved by detecting the absence of *SMN1* exon 7.

The development of disease-modifying therapies, including nusinersen, onasemnogene abeparvovec, and risdiplam, has significantly improved the prognosis of SMA [6,7]. Importantly, accumulating evidence shows that treatment initiated before symptom onset has the greatest clinical benefit. Consequently, to enable presymptomatic diagnosis and early therapeutic intervention, newborn screening (NBS) for SMA has been implemented in several countries, including Japan.

The current SMA-NBS programs are primarily based on the results of real-time polymerase chain reaction (PCR) assay, which detects homozygous deletion of *SMN1* exon 7 from dried blood spot (DBS) specimens. This assay can successfully identify the vast majority of patients with SMA. However, despite having SMA, individuals with compound heterozygous *SMN1* variants who retain one copy of *SMN1* exon 7 may not be identified by standard screening methods. Therefore, they can receive false-negative screening results.

This study was conducted before the implementation of population-based SMA-NBS in Tokyo to evaluate the analytical performance of the real-time PCR-based assay planned for use in the Tokyo screening program. In addition, we evaluated a patient with compound heterozygous *SMN1* variants who had previously received a false-negative SMA-NBS result in another prefecture, to confirm the known limitation of deletion-based screening using this assay.

## 2. Materials and Methods

### 2.1. Study Design

This study was conducted between December 2022 and March 2023, before the implementation of population-based SMA-NBS in Tokyo, to evaluate the analytical performance of the real-time PCR-based assay planned for use in the Tokyo screening program. The study was not designed to estimate the diagnostic sensitivity or specificity of population-based SMA-NBS.

### 2.2. Participants

Newborns born at the National Center for Child Health and Development who underwent DBS collection for Pompe disease NBS, which is a part of the institution’s optional expanded NBS program, were eligible for inclusion in this study. Parents or legal guardians received information regarding the study and provided written informed consent before participation.

In addition, three patients with genetically confirmed SMA who were receiving follow-up care at the National Center for Child Health and Development were enrolled as positive controls after separate informed consent was obtained. These included two patients with homozygous *SMN1* deletions and one with compound heterozygous *SMN1* variants comprising a deletion on one allele and a pathogenic sequence variant on the other allele. At the time of blood sampling, the two patients with homozygous *SMN1* deletion were both 7 years old, whereas the patient with compound heterozygous *SMN1* variants was 3 months old.

### 2.3. Sample Collection

For newborn participants, a portion of the DBS specimens collected for Pompe disease NBS was used for SMA testing. For the three SMA control patients, approximately 50 µL of peripheral blood obtained during routine clinical visits was applied to filter paper to prepare an approximately 1-cm-diameter DBS, as for the newborn specimens. The control DBS specimens were dried in the same room and under the same conditions as the newborn specimens. For SMA testing, a 1.5-mm-diameter disc was punched from each DBS and analyzed in the same manner for both newborn and control specimens. The DBS specimen from the patient with compound heterozygous *SMN1* variants was prepared from peripheral blood obtained after the diagnosis of SMA but before gene therapy and was not the original neonatal screening specimen.

All specimens were assigned anonymized study identification numbers prior to analysis. Personal identifiers were removed from the analytical dataset.

### 2.4. SMA-NBS Assay

DBS specimens were analyzed using the NeoSMAAT^®^ T/K/S assay kit (Sekisui Medical Co., Ltd., Tokyo, Japan), a real-time PCR-based assay designed for NBS. The assay simultaneously detects *SMN1*, T-cell receptor excision circles, kappa-deleting recombination excision circles, and RNase P as an internal control. RNase P was used to assess specimen adequacy and amplification quality; however, the *SMN1* copy-number value was not normalized to the RNase P value. The assay also enables direct amplification from DBS punches without prior DNA extraction. The assay was performed according to the manufacturer’s instructions. Supplementary Method S1 shows the detailed assay procedures and PCR conditions [8].

Except for the compound heterozygous SMA sample, each specimen was analyzed once. For the compound heterozygous sample, two 1.5-mm punches were obtained from each of two separate DBSs and analyzed individually (four measurements in total). According to the manufacturer’s reference data, the criterion for RNase P was ≥668 copies/µL of whole blood. The laboratory recorded no internal-control failures requiring repeat testing, although individual RNase P values were not retained in the dataset used for this study.

### 2.5. Data Analysis

*SMN1* exon 7 copy number was quantified according to the manufacturer’s protocol. The reported *SMN1* values are expressed as copies per microliter of whole blood. According to the manufacturer’s calculation method, a 1.5-mm DBS punch was considered to contain 0.75 µL of whole blood, and the PCR-derived concentration was converted to copies per microliter of whole blood using this conversion factor. For this study, the Tokyo Health Service Association set the screening cutoff for *SMN1* at 668 copies/µL based on the manufacturer’s reference data. The measured *SMN1* copy numbers of newborn participants and SMA control subjects were compared descriptively.

## 3. Results

### 3.1. Study Participants

Table 1 shows the characteristics of the participants. In total, 21 newborns were enrolled during the study period, and they underwent SMA-NBS. All participants were born at term (≥37 weeks of gestation). The newborns who were enrolled had no family history of SMA. None of the participants developed clinical features indicative of SMA during at least 18 months of follow-up, as ascertained by review of the medical records.

Three patients with genetically confirmed SMA were included as positive controls. Two patients had SMA type 2 caused by homozygous *SMN1* deletion (*SMN1*:c.[0]) and had not undergone NBS. The remaining patient was diagnosed with SMA type 1 caused by compound heterozygous *SMN1* variants comprising a deletion on one allele and a pathogenic sequence variant on the other allele (*SMN1*:c.[294G>A];[0]). This patient had undergone SMA-NBS at birth in another prefecture, where SMA-NBS had already been implemented, and had received a negative screening result. However, muscle weakness developed within the first month of life, and subsequent genetic testing confirmed the diagnosis of SMA. The clinical course and genetic findings of this same patient have been reported previously [9].

### 3.2. SMN1 Copy Number Analysis

**Figure 1:**
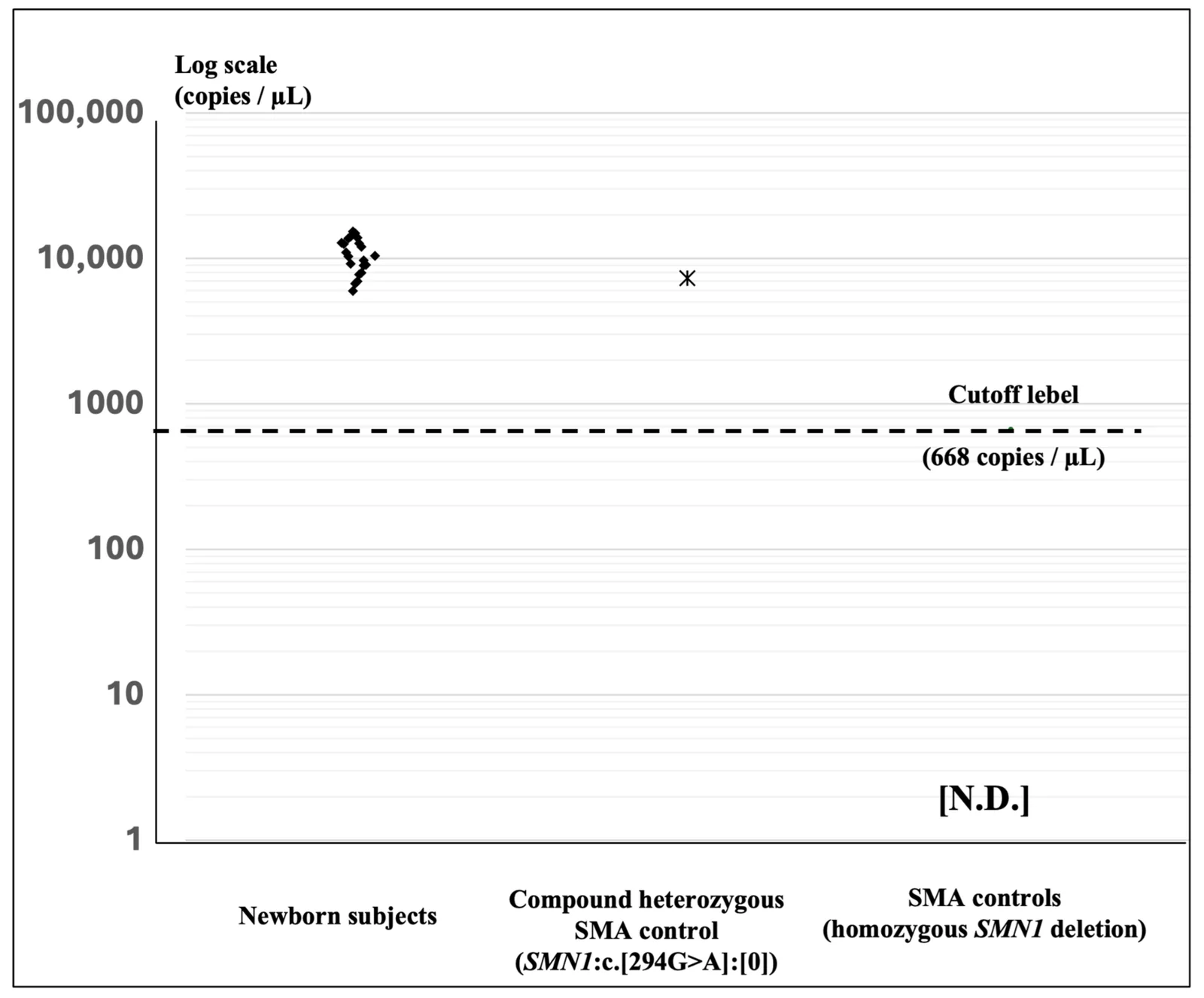
*SMN1* exon 7 copy numbers were measured using the SMA newborn screening assay.

The predefined screening cutoff value of the assay was 668 copies/µL. The mean *SMN1* exon 7 copy number of the newborn participants (n = 21) was 10,750 ± 2840 copies/µL (mean ± standard deviation), with values ranging from 5970 to 15,300 copies/µL (Table 2). All newborn samples were above the screening cutoff and were classified as screen-negative. The two SMA control patients with homozygous *SMN1* deletion did not present with detectable *SMN1* amplification, which is consistent with a positive screening result. In contrast, for the SMA control patient with compound heterozygous *SMN1* variants, four measurements were performed using two 1.5-mm punches from each of two separate DBSs. All four measurements exceeded the screening cutoff by more than 10-fold, with a mean *SMN1* value of 7330 copies/µL. The mean value was within the range observed in the newborn cohort (Figure 1).

The *SMN1* copy numbers of the newborn screening participants (n = 21), a patient with compound heterozygous SMA carrying *SMN1*:c.[294G>A];[0], and two patients with SMA who presented with homozygous *SMN1* deletion are shown. The dashed horizontal line indicates the screening cutoff value (668 copies/µL). The patient with compound heterozygous SMA showed an *SMN1* copy number above the cutoff value despite having genetically confirmed SMA and having received a negative newborn screening result. Each dot represents an individual newborn participant, and the asterisk (*) indicates the patient with compound heterozygous SMN1 variants. N.D., Not Detected.

## 4. Discussion

In this pre-implementation study, we confirmed the known false-negative limitation of deletion-based SMA-NBS using a DBS sample from a patient with compound heterozygous *SMN1* variants who had actually received a negative SMA-NBS result. When analyzed using the assay planned for use in the Tokyo screening program, the *SMN1* exon 7 signal from this patient fell within the range observed in unaffected newborns. Thus, the present study provides analytical confirmation of this limitation using a clinically documented false-negative case.

The current SMA-NBS programs primarily target homozygous deletion of *SMN1* exon 7 using real-time PCR-based assays [10,11]. Approximately 95% of patients with SMA carry homozygous *SMN1* deletions. Hence, these programs achieve excellent detection rates, and they have enabled presymptomatic treatment for several affected infants [4,11]. However, about 5% of patients with SMA harbor compound heterozygous *SMN1* variants comprising a deletion on one allele and an intragenic pathogenic variant on the other allele [1,4,11]. In such cases, at least one copy of *SMN1* exon 7 remains detectable, resulting in a normal screening finding despite the presence of disease-causing variants. Similar false-negative cases have been reported in other NBS programs [11,12], indicating that this limitation is inherent to deletion-based screening approaches rather than to a particular assay platform. A recent systematic review identified six reported false-negative cases in 37 SMA-NBS studies, including three patients with compound heterozygous *SMN1* variants [11]. The compound heterozygous patient evaluated in the present study was previously reported as a clinical case [9]. The present study extends that report by analytically evaluating a DBS sample from this patient using the assay planned for implementation in the Tokyo screening program.

Our results further show that samples retaining one detectable copy of *SMN1* exon 7 cannot be reliably distinguished from normal samples using the screening approach used in this study. The assay was designed to detect the absence of *SMN1* exon 7 rather than to determine *SMN1* allelic copy number. In the newborn cohort, the mean *SMN1* value was 10,750 copies/µL, with a standard deviation of 2840 copies/µL (26.4% of the mean) and a range of 5970–15,300 copies/µL. The value in the patient with compound heterozygous *SMN1* variants was 7330 copies/µL, which was within the range observed in the newborn cohort. RNase P was used as an internal control but was not used to normalize the *SMN1* copy-number value. Because amplification was performed directly from a DBS punch without prior DNA extraction, the absolute *SMN1* value may also be influenced by the amount of blood and cellular material contained in each punch. These characteristics, together with variability inherent to DBS specimens, indicate that defining a reliable quantitative threshold to distinguish one from two *SMN1* copies would not be appropriate using this assay. The assay is not designed to determine carrier status and should not be considered a carrier screening test [10].

Several approaches may be considered to reduce the risk of false-negative results in deletion-based SMA-NBS. Second-tier *SMN1* sequencing could theoretically identify pathogenic sequence variants in samples retaining *SMN1* exon 7; however, applying sequencing to a large number of screen-negative newborns would increase cost, turnaround time, and laboratory workload. Lowering the screening cutoff to identify newborns with a single detectable *SMN1* copy would also result in the identification of a large number of carriers, given the carrier frequency of approximately 1 in 50 [1], and would therefore not be practical for population-based screening. In contrast, when SMA is clinically suspected in an infant with hypotonia or muscle weakness, a negative NBS result should not delay diagnostic genetic testing. In such cases, comprehensive *SMN1* testing, including deletion/duplication analysis by MLPA and sequencing for intragenic pathogenic variants, should be considered.

The clinical implication of this study is that a negative SMA-NBS result does not completely exclude the SMA diagnosis. Clinicians should continue to consider SMA in infants presenting with hypotonia, muscle weakness, feeding difficulties, or delayed motor development despite a negative screening result [11,12]. In particular, early diagnosis remains important because the currently available disease-modifying therapies have the greatest benefit when initiated as early as possible [6,7].

This study has several limitations. First, the number of newborn participants and SMA control patients was small, and only a single patient with compound heterozygous *SMN1* variants was evaluated. Second, the study was designed to evaluate an analytical limitation of the screening assay rather than to estimate the sensitivity or specificity of a population-based screening program. Third, the DBS specimens from the SMA control patients were prepared from peripheral venous blood, whereas the newborn DBS specimens were collected by heel prick. Differences in blood source and DBS preparation may affect hematocrit and blood distribution on filter paper and, consequently, the absolute *SMN1* copy-number values obtained from a fixed-size DBS punch. In addition, the compound heterozygous sample was collected at three months of age, and differences in leukocyte content between neonatal and later-infant blood samples may have affected the amount of genomic DNA available for direct amplification. However, the control DBS specimens were dried under the same conditions as the newborn specimens, and the same 1.5-mm punch procedure was used for all specimens. Therefore, although direct quantitative comparison of absolute *SMN1* copy-number values between the two specimen types should be interpreted with caution, we consider it unlikely that these pre-analytical differences substantially affected the qualitative screening classification of the compound heterozygous sample. Fourth, except for the compound heterozygous SMA sample, each specimen was analyzed only once; therefore, intra-assay variability and reproducibility could not be evaluated for these single-measurement specimens. Although the compound heterozygous sample was measured four times, only the mean value was retained in the study dataset, and the individual measurement values were unavailable for assessment of variability. Larger-scale studies are needed to further characterize the frequency and clinical impact of false-negative results in patients with non-deletion *SMN1* variants [11].

## 5. Conclusions

In this study, a patient with compound heterozygous *SMN1* variants showed an *SMN1* exon 7 copy number within the normal screening range. This patient would not have been identified by a deletion-based SMA-NBS assay. These findings provide analytical confirmation of an inherent false-negative limitation of the current SMA-NBS programs and emphasize that a negative screening result does not completely rule out SMA. Clinical suspicion and confirmatory molecular testing remain important if SMA is suspected.

## Figures and Tables

**Table 1:** Participant Characteristics.

Group	Participants
Newborn screening participants	21 (Male: 9, Female: 12)
SMA control patients with homozygous *SMN1* deletion (*SMN1*:c.[0])	2 (Male: 1, Female: 1)
SMA control patient with compound heterozygous *SMN1* variants (*SMN1*:c.[294G>A];[0])	1 (Male: 1)

Abbreviations: SMA, spinal muscular atrophy; NBS, newborn screening.

**Table 2:** Results of real-time PCR analysis of the 21 newborn participants.

	Copies/µL
Number of Specimens	21 (Male: 9, Female: 12)
Mean	10,750
Standard Deviation	2840
Minimum Value	5970
Median	10,400
Maximum Value	15,300

## Data Availability

The individual *SMN1* values of the 21 newborn participants are provided in Supplementary Table S1. Additional data presented in this study are available from the corresponding author upon reasonable request.

## Supplementary Materials

The following supporting information can be downloaded at: https://www.mdpi.com/article/10.3390/ijns12030071/s1.

## Abbreviations

The following abbreviations are used in this manuscript:

SMA	Spinal muscular atrophy
NBS	Newborn screening
DBS	Dried blood spot
PCR	Polymerase Chain Reaction
