# Supplementary material for: Analytical Study Showing a False-Negative Limitation of Deletion-Based Newborn Screening for Spinal Muscular Atrophy Using a Compound Heterozygous SMN1 Control
Source: Int J Neonatal Screen. 2026 Aug 31;12(3):71. doi: 10.3390/ijns12030071 (PMC13607524; doi:10.3390/ijns12030071)
Supplement: Supplementary file 1 [file IJNS-12-00071-s001.zip › IJNS-4425953-supplementary.pdf]

S1. Supplementary Methods

The NeoSMAAT® T/K/S assay (Sekisui Medical Co., Ltd., Tokyo, Japan) was performed according to the manufacturer's instructions. The assay utilizes a 1.5-mm dried blood spot (DBS) punch and allows direct PCR amplification without prior DNA extraction.
PCR amplification was performed under the following conditions:

1. Initial denaturation:
   - 95° C for 15 min (1 cycle)
2. Pre-amplification:
   - 95° C for 15 s and 63° C for 80 s
   - 20 cycles without fluorescence acquisition
3. Quantitative amplification:
   - 95° C for 15 s and 63° C for 80 s
   - 25 cycles with fluorescence acquisition

Further details of the assay procedure are available in the manufacturer's instructions for use.

Reference:
Sekisui Medical Co., Ltd. NeoSMAAT® T/K/S Instructions for Use. Available at: https://www.sekisuimedical.jp/business/diagnostics/others/neosmaat_tks/operate.html (Accessed June 12, 2026).

Supplementary Table S1

Participant	*SMN1* (copies/μL)
1	12737
2	9695
3	9006
4	7953
5	12038
6	14847
7	12563
8	10443
9	10273
10	6700
11	6930
12	12663
13	7747
14	8905
15	13810
16	10965
17	9176
18	15334
19	5971
20	13717
21	14131
